# Supplementary material for: Citrus Bright Spot Virus: A New Dichorhavirus, Transmitted by Brevipalpus azores, Causing Citrus Leprosis Disease in Brazil
Source: Plants (Basel). 2023 Mar 20;12(6):1371. doi: 10.3390/plants12061371 (PMC10053991; doi:10.3390/plants12061371)
Supplement: Supplementary file 1 [file plants-12-01371-s001.zip › Supplementary Figure S1.pdf]

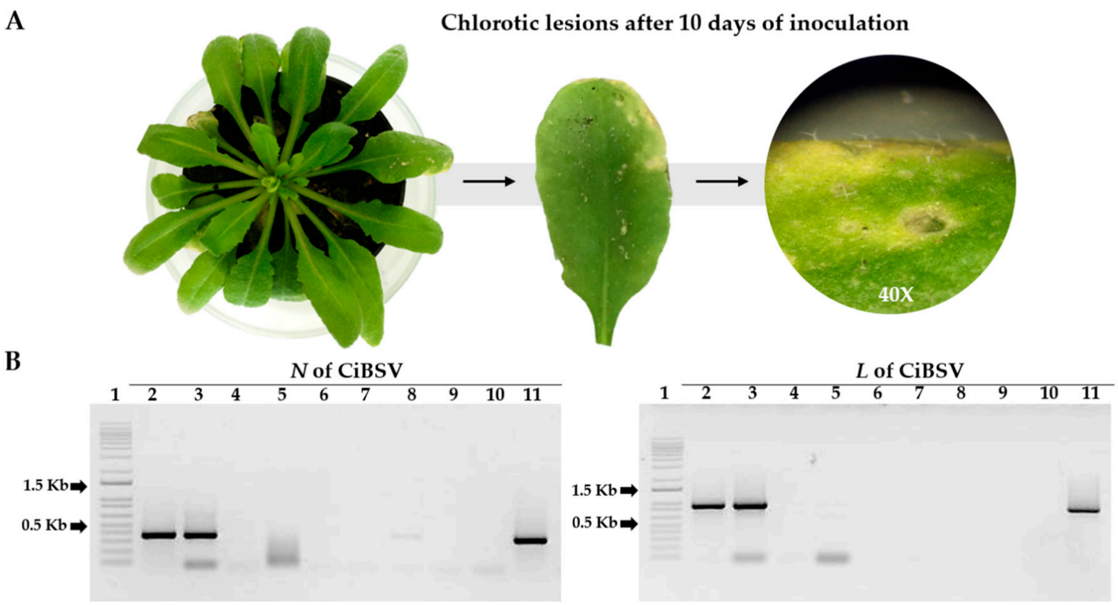

Figure S1: Experimental transmission of CiBSV to *Arabidopsis thaliana* Col-0 mediated by *Brevipalpus azores*. (A) Symptomatic leaves of Arabidopsis plants after 10 days inoculated with *B. azores* mites found in a symptomatic sweet orange (*Citrus sinensis*) tree collected in Passo Fundo (PFd), RS, Brazil, 2019. (B) The 1% agarose gel electrophoresis of RT-PCR products using specific pair primers for detection of the *N* (296 bp) e *L* (938 bp) genes of CiBSV. Lane 1: molecular weight marker, 1 Kb Plus DNA Ladder (Thermo Fisher Scientific, Madison, WI, USA); lanes 2-8: symptomatic leaves of Arabidopsis after 10 days of transmission; lane 9: Reverse-transcription blank; lane 10: PCR blank; lane 11: CiBSV-infected sweet orange from PFd.
