# Supplementary material for: Citrus Bright Spot Virus: A New Dichorhavirus, Transmitted by Brevipalpus azores, Causing Citrus Leprosis Disease in Brazil
Source: Plants (Basel). 2023 Mar 20;12(6):1371. doi: 10.3390/plants12061371 (PMC10053991; doi:10.3390/plants12061371)
Supplement: Supplementary file 1 [file plants-12-01371-s001.zip › Supplementary Figure S2.pdf]

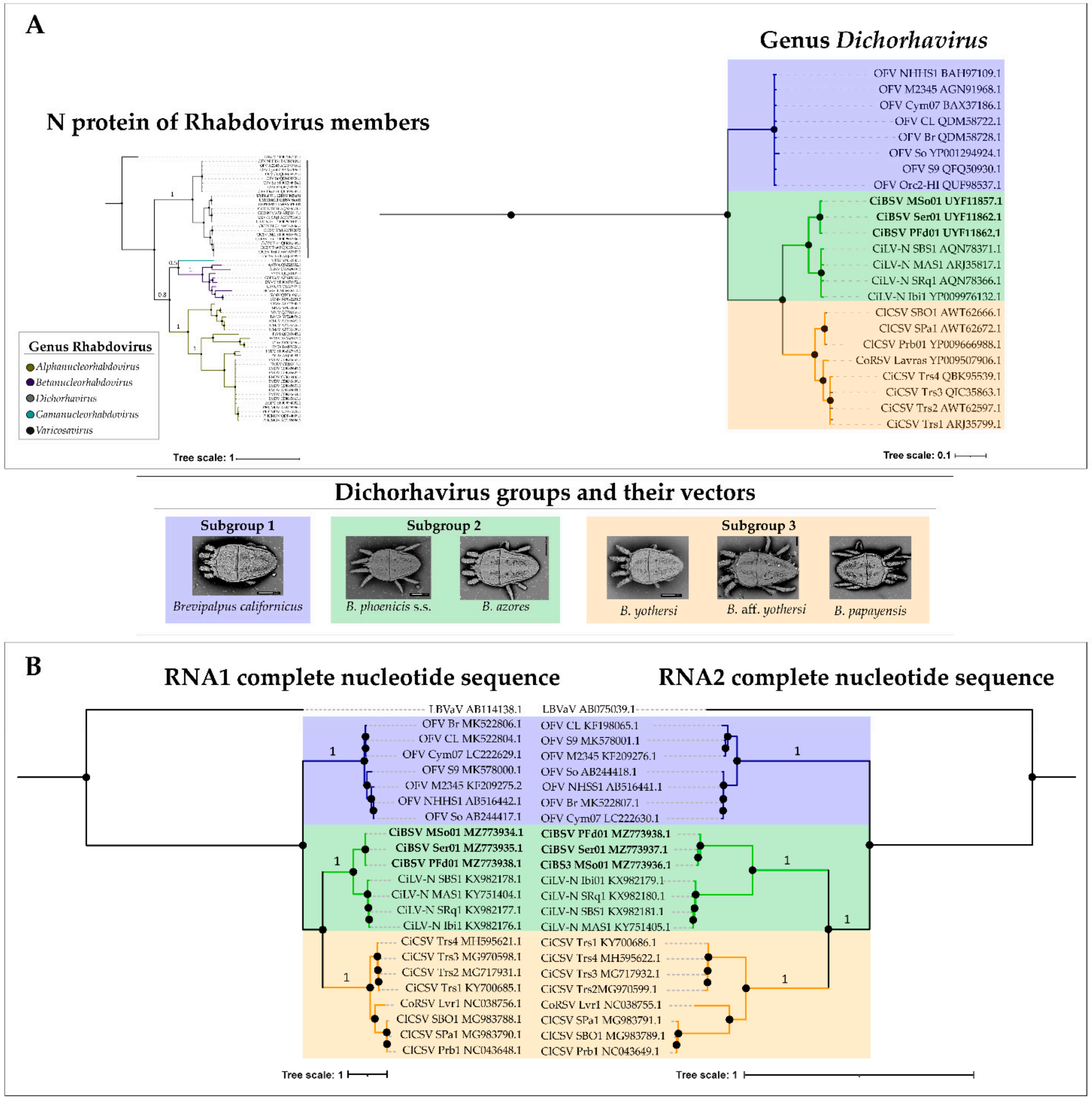

A
N protein of Rhabdovirus members
Genus Rhabdovirus
Alphanucleorhabdovirus
Betanucleorhabdovirus
Dichorhavirus
Gamanucleorhabdovirus
Varicosavirus
Tree scale: 1
Genus Dichorhavirus
OFV NHHS1 BAH97109.1
OFV M2345 AGN91968.1
OFV Cym07 BAX37186.1
OFV CL QDM58722.1
OFV Br QDM58728.1
OFV So YP001294924.1
OFV S9 QFQ50930.1
OFV Orc2-HI QUF98537.1
CiBSV MSo01 UYF11857.1
CiBSV Ser01 UYF11862.1
CiBSV PFd01 UYF11862.1
CiLV-N SBS1 AQN78371.1
CiLV-N MAS1 ARJ35817.1
CiLV-N SRq1 AQN78366.1
CiLV-N Ibi1 YP009976132.1
ClCSV SBO1 AWT62666.1
ClCSV SPa1 AWT62672.1
ClCSV Prb01 YP009666988.1
CoRSV Lavras YP009507906.1
CiCSV Trs4 QBK95539.1
CiCSV Trs3 QIC35863.1
CiCSV Trs2 AWT62597.1
CiCSV Trs1 ARJ35799.1
Tree scale: 0.1
Dichorhavirus groups and their vectors
Subgroup 1
Brevipalpus californicus
Subgroup 2
B. phoenicis s.s.
B. azores
Subgroup 3
B. yothersi
B. aff. yothersi
B. papayensis
B
RNA1 complete nucleotide sequence
RNA2 complete nucleotide sequence
LBVaV AB114138.1
OFV Br MK522806.1
OFV CL MK522804.1
OFV Cym07 LC222629.1
OFV S9 MK578000.1
OFV M2345 KF209275.2
OFV NHHS1 AB516442.1
OFV So AB244417.1
CiBSV MSo01 MZ773934.1
CiBSV Ser01 MZ773935.1
CiBSV PFd01 MZ773938.1
CiLV-N SBS1 KX982178.1
CiLV-N MAS1 KY751404.1
CiLV-N SRq1 KX982177.1
CiLV-N Ibi1 KX982176.1
CiCSV Trs4 MH595621.1
CiCSV Trs3 MG970598.1
CiCSV Trs2 MG717931.1
CiCSV Trs1 KY700685.1
CoRSV Lvr1 NC038756.1
ClCSV SBO1 MG983788.1
ClCSV SPa1 MG983790.1
ClCSV Prb1 NC043648.1
LBVaV AB075039.1
OFV CL KF198065.1
OFV S9 MK578001.1
OFV M2345 KF209276.1
OFV So AB244418.1
OFV NHSS1 AB516441.1
OFV Br MK522807.1
OFV Cym07 LC222630.1
CiBSV PFd01 MZ773938.1
CiBSV Ser01 MZ773937.1
CiBS3 MSo01 MZ773936.1
CiLV-N Ibi01 KX982179.1
CiLV-N SRq1 KX982180.1
CiLV-N SBS1 KX982181.1
CiLV-N MAS1 KY751405.1
CiCSV Trs1 KY700686.1
CiCSV Trs4 MH595622.1
CiCSV Trs3 MG717932.1
CiCSV Trs2MG970599.1
CoRSV Lvr1 NC038755.1
ClCSV SPa1 MG983791.1
ClCSV SBO1 MG983789.1
ClCSV Prb1 NC043649.1
Tree scale: 1
Tree scale: 1

Figure S2: Phylogenetic reconstruction of the members of the genus *Dichorhavirus* and CiBSV isolates (bold). Bayesian inferences were based on the amino acid sequence of N protein (A) and the complete sequence of RNA1 and RNA2 (B). The phylogenetic trees were generated using MrBayes with 2,000,000 generations. and the varicosavirus lettuce big-vein associated virus (LBVaV) was used as an outgroup in the trees from amino acid sequences. The colors of branches are based on the dichorhavirus subgroup, according to the vector *Brevipalpus* species: subgroup 1 (blue; transmitted by *Brevipalpus californicus*), subgroup 2 (green; transmitted by *B. phoenicis* s.s. and *B. azores*), and subgroup 3 (orange; transmitted by *B. papayensis, B. yothersi,* and *B.* aff. *yothersi*);
