# Supplementary material for: Citrus Bright Spot Virus: A New Dichorhavirus, Transmitted by Brevipalpus azores, Causing Citrus Leprosis Disease in Brazil
Source: Plants (Basel). 2023 Mar 20;12(6):1371. doi: 10.3390/plants12061371 (PMC10053991; doi:10.3390/plants12061371)
Supplement: Supplementary file 1 [file plants-12-01371-s001.zip › Supplementary Table S1.pdf]

Table S1: List of primers used for validation and obtaining the 3′ and 5′ extremes of the citrus bright spot virus genomes.

Sequences (5′—3′)	Direction	Target Region[1]	Size (bp)
**RNA1**			
CAGTCACTATAGATTACTCAGCAG	F:	265-288	945
CTCTGCTGTACCTCCAGAAT	R:	1190-1209	
AAACATACATGGACCACTCA	F:	977-996	938
TCATAGGTGGTGTTATTCCA	R:	1895-1914	
AGCATGATCGGTACATCAA	F:	1770-1788	706
AGTTCAGGCATGGCTTCT	R:	2458-2475	
AAGCATTCTCCAGGAAGAC	F:	2227-2245	942
GTTGGTGACAGCCTTGTT	R:	3151-3168	
CAACAAGACACTCCAAACTG	F:	2901-2920	882
ATATATGTGAACTTCTTGTCACC	R:	3760-3782	
TTCAAGGGTTCACAGATATAGA	F:	3533-3554	937
CGACAGTGGCACTTATTTC	R:	4451-4469	
AGAGGTGGCCAAATTCTC	F:	4222-4239	945
CTGCATCTGTTGTACTGGAG	R:	5147-5166	
ATGAGTATAGTCCCCAACACAG	F:	4917-4938	930
GAGGATGATGGTGCTGAC	R:	5829-5846	
GGAGAGTCTTCTAGCAAAACAG	F:	5598-5619	852
CTCCATAAGGCTTGCACTA	R:	6431-6449	
**RNA2**			
TCTCTTCTTGATTCTTCTTTCA	F:	201-222	926
CCTGTCACCTCATCTACCAT	R:	1107-1126	
CAATATGATTGTGTACAACAGG	F:	884-905	940
GATGGTAAAGTACAGCCTCA	R:	1804-1823	
TCAGAACTAATACAGATGATCAGG	F:	1581-1604	940
ATGTTACATTGTTAAAATACATCATCT	R:	2494-2520	
ATGAAGGTGGACCTTGTG	F:	2271-2288	931
TATCACCGGAGCAGAGAC	R:	3184-3201	
AAGGAGCCCAAATACCTG	F:	2952-2969	940
GTATCGAGTATGCAATGTTTG	R:	3871-3891	
GCCTACGGAGAGGAAGAT	F:	3654-3671	938
GGGTCTACTGAGCTGTATATGA	R:	4570-4591	
GCTGATTACTTAAGCATGGA	F:	4341-4360	935
GAGCCACATCTCTCGTACA	R:	5257-5275	
TATGGCGGAACTTCTATATG	F:	5038-5057	780
TTAGCAATATGAACCCCATC	R:	5798-5817	
**RACE 5′/3′ universal primer Clontech®**			
TAATACGACTCACTATAGGGCAAGCAGTGGTATCAA CGCAGAGT	*Long*	-	-
CTAATACGACTCACTATAGGGC	*Short*	-	-
**RNA1 5′ RACE**			

GATTACGCCAAGCTTCGTTAGCAGCTGCAGCCC TGGAGTC	F *Long*	612-636	636
TGCTACTCCTACCATCAC	F *Short*	543-560	560
**RNA1 3′ RACE**			
GATTACGCCAAGCTTTGACACCTCTTGCATACCGAG CCTTTCC	F *Long*	5939-5966	718
TCAATGTTGAGGTCAGTAG	F *Short*	6210-6228	447
**RNA2 5′ RACE**			
GATTACGCCAAGCTTGTCGAGTCATCGTTGCGATAG AGTTCCTGGATG	F *Long*	485-517	517
TGCCAGCAGTTTACATG	F *Short*	409-425	425
**RNA2 3′ RACE**			
GATTACGCCAAGCTTCTGTCCCTGGCCATAGCACTTT TCCACTC	F *Long*	5460-5488	552
TGTGAGGAGCCTGTTC	F *Short*	5534-5549	478

[1] Based on CiBSV_PFd01 genome sequence (GenBank accession number of RNA1: MZ773933 and RNA2: MZ773938).
