# Supplementary material for: Citrus Bright Spot Virus: A New Dichorhavirus, Transmitted by Brevipalpus azores, Causing Citrus Leprosis Disease in Brazil
Source: Plants (Basel). 2023 Mar 20;12(6):1371. doi: 10.3390/plants12061371 (PMC10053991; doi:10.3390/plants12061371)
Supplement: Supplementary file 1 [file plants-12-01371-s001.zip › Supplementary Table S2.pdf]

Table S2: Percentage of nucleotide (nt) and deduced amino acid (aa) among CiBSV isolates and other members of the genus *Dichorhavirus*.

**CiBSV PFd01**[1]	**CiBSV MSa01**		**CiBSV Ser01**		**CiLV-N**		**OFV**		**CoRSV**		**ClCSV**		**CiCSV**	
	**nt**	**aa**	**nt**	**aa**	**nt**	**aa**	**nt**	**aa**	**nt**	**aa**	**nt**	**aa**	**nt**	**aa**
RNA1	98	-	97	-	68		47	-	51	-	51	-	51	-
N	98	99	98	99	74	81	54	50	60	58	60	58	59	58
P	99	99	99	99	71	75	48	33	55	47	56	47	55	45
MP	98	100	98	100	78	85	63	63	64	63	64	64	64	62
M	98	98	97	98	69	73	49	40	55	50	55	47	57	49
G	98	98	98	98	70	74	43	33	48	40	48	38	48	39
RNA2	98	-	98	-	74	-	57	-	60	-	60	-	60	-
L	98	99	98	99	74	83	59	57	62	64	63	64	63	64

[1]Virus name acronyms: citrus bright spot virus (CiBSV), isolates Passo Fundo 01 (PFd01), Marquês de Souza 01 (MSo01) and Seara 01 (Ser01); citrus leprosis virus N (CiLV-N); orchid fleck virus (OFV); coffee ringspot virus (CoRSV); clerodendrum chlorotic spot virus (ClCSV) and citrus chlorotic spot virus (CiCSV).
