# Supplementary material for: Citrus Bright Spot Virus: A New Dichorhavirus, Transmitted by Brevipalpus azores, Causing Citrus Leprosis Disease in Brazil
Source: Plants (Basel). 2023 Mar 20;12(6):1371. doi: 10.3390/plants12061371 (PMC10053991; doi:10.3390/plants12061371)
Supplement: Supplementary file 1 [file plants-12-01371-s001.zip › Supplementary Table S3.pdf]

Table S3: Annotations of conserved domains, signal peptides, and transmembrane domains found in the putative proteins of CiBSV

**Prediction of conserved domains using MotifFinder[1] program**			
**Protein/isolate[5]**	**Pfam**	**Amino acid position (E-value)**	**Description**
N/CiBSV_PFd01	Rhabdo_ncap_2	127..338 (4.9e-22)	PF03216, Rhabdovirus nucleoprotein
N/CiBSV_MSa01	Rhabdo_ncap_2	127..338 (7.5e-22)	PF03216, Rhabdovirus nucleoprotein
N/CiBSV_Ser01	Rhabdo_ncap_2	127..338 (1.1e-21)	PF03216, Rhabdovirus nucleoprotein
P/CiBSV_PFd01	DUF5856	93..146 (0.41)	PF19174, Family of unknown function (DUF5856)
P/CiBSV_PFd01	DUF287	132..152 (0.3)	PF03384, Drosophila protein of unknown function, DUF287
P/CiBSV_MSa01	DUF5856	93..146 (0.43)	PF19174, Family of unknown function (DUF5856)
P/CiBSV_Ser01	DUF5856	93..146 (0.43)	PF19174, Family of unknown function (DUF5856)
G/CiBSV_PFd01	UL11	213..228 (0.08)	PF11094, Membrane-associated tegument protein
L/CiBSV_MSa01	Mononeg_RNA_pol	141..1042 (6.7e-162)	PF00946, Mononegavirales RNA dependent RNA polymerase
L/CiBSV_MSa01	Mononeg_mRNAcap	1073..1290 (1.1e-37)	PF14318, Mononegavirales mRNA-capping region V
L/CiBSV_Ser01	Mononeg_RNA_pol	154..1042 (1e-161)	PF00946, Mononegavirales RNA dependent RNA polymerase
L/CiBSV_Ser01	Mononeg_mRNAcap	1073..1290 (1.1e-37)	PF14318, Mononegavirales mRNA-capping region V
L/CiBSV_PFd01	Mononeg_RNA_pol	136..1042 (1e-161)	PF00946, Mononegavirales RNA dependent RNA polymerase
L/CiBSV_PFd01	Mononeg_mRNAcap	1073..1290 (2e-36)	PF14318, Mononegavirales mRNA-capping region V
**Prediction of signal peptides using SignalP-5.0 program[2]**			
	**Cleavage site between position**	**Signal Peptide (Sec/SPI)**	

G/CiBSV_PFd01	25 and 26	0.9481
G/CiBSV_MSa01		0.9474
G/CiBSV_Ser01		0.9374
**Prediction of transmembrane helice using TMHMM - 2.0 program[3]**		
	**Amino acid position**	**Probability**
G/CiBSV_PFd01	493-500	≥0.99≤1
G/CiBSV_MSa01	492-500	≥0.99≤1
G/CiBSV_Ser01	493-500	≥0.99≤1
**Prediction of eukaryotic protein subcellular localization using Deeploc program[4]**		
	**Probability of localization in cell membrane**	
G/CiBSV_PFd01	0.9181	
G/CiBSV_MSa01	0.8268	
G/CiBSV_Ser01	0.9094	

Used programs for annotation: [1]MOTIF Search (https://www.genome.jp/tools/motif/), [2]SignalP-5.0 (Petersen et al., 2011), [3]TMHMM Server version 2.0 (Krogh et al., 2001), and [4]Deeploc version 17 (Almagro Armenteros et al., 2017); [5]Putative proteins of citrus bright spot virus isolate PaF01 (collected in Passo Fundo, RS), MSa01 (Marquês de Souza, RS) and Ser01 (Seara, SC): Nucleocapsid (N); Phosphoprotein (P); Glycoprotein (G) and RNA-dependent RNA polymerase (L).
